# Inspiring hope, confronting hopelessness: healthcare experiences of Black/African American pregnant and post-partum women and healthcare workers in Detroit, Michigan, U.S.A.

**DOI:** 10.1186/s12884-026-08666-5

**Published:** 2026-01-27

**Authors:** Linda Kaljee, Doreen Dankerlui, Alfred Pach, Brent Davidson, Ijeoma Nnodim Opara, Linda Reyes, Lisa MacLean

**Affiliations:** 1 Global Health Initiative, Henry Ford Health, Detroit, Michigan USA; 2 Women’s Health, Henry Ford Health, Detroit, MI USA; 3 FAAP, Wayne State University, Detroit, MI USA; 4 CHW, Office of Community Health, Wellness and Diversity, Henry Ford Health, Detroit, MI USA; 5 Behavioral Health Services, Henry Ford Health, Detroit, MI USA

**Keywords:** Hope, Black/African american, Pregnancy, Post-partum, Healthcare workers, Burn-out.

## Abstract

**Background:**

The United States has the highest maternal mortality rate among high-income countries. Black/African American women and infants experience significant disparities in mortality rates. Henry Ford Global Health Initiative implemented a mixed-methods study to explore the association of hopefulness and burn out among healthcare workers (HCWs) and health experiences and outcomes of Black/African American pregnant and post-partum healthcare recipients (HCRs).

**Methods:**

Quantitative data included demographics and the Herth Hope Index (HHI). Qualitative research included focus groups and individual interviews with HCWs and HCRs from two Henry Ford Health Women’s Health clinics. Prior to data collection, a literature review was completed. Descriptive data and bivariate analysis were completed. Qualitative data used semi-structured interview guides and were audio-taped, transcribed, and coded using Dedoose. Final data tables included codes, sub-themes, and representative quotes. Stakeholder workshops were held in 2020 and 2024.

**Results:**

Sixty-six HCWs completed the demographic form and HHI. In 2021, 34 (87.2%) HCWs participated in focus groups or interviews and 25 (92.6%) in 2023. Sixty-one HCRs completed the demographic form and HHI and 27 (44.3%) participated in focus groups. On the 48-point HHI scale, HCWs scored 41.7 (SD 4.6) and HCRs scored 40.8 (SD 7.0) indicating a relatively high level of hopefulness. HCW qualitative outcomes included: (1) definitions of hopefulness and hopelessness: (2) personal and work-related sources of care and support; and (3) clinical and systemic challenges and facilitators to meeting patient needs. HCR qualitative outcomes included: (1) definitions of hopefulness and hopelessness; (2) personal, familial, and social sources of care and support; and (3) pregnancy and post-partum healthcare experiences. In 2020, workshop participants generated factors affecting hope in both HCRs and HCWs and potential solutions to reduce burnout and improve pre- and post-partum care for Black/African American women. In 2024, workshop participants reviewed data and prioritized recommendations for interventions.

**Conclusion:**

This study explored the role of hope as a contributing factor in the experiences of HCWs and Black/African American pregnant and post-partum HCRs. It generated recommendations to address hopelessness and support positive experiences and outcomes. Next steps include identifying resources to pilot and evaluate recommendations at the health system and clinical levels.

**Supplementary Information:**

The online version contains supplementary material available at 10.1186/s12884-026-08666-5.

## Introduction

The United States has the highest maternal mortality rate among high-income countries with Black/African American women experiencing a three-fold higher rate of death than White women [1, 2]. In addition, the infant mortality rate for Black/African American infants is 10.9/1000 compared to 4.5/1000 for White infants [3]. Black/African American women are also approximately 50% more likely to have preterm deliveries than White or Latina women [4].

While over 60% of maternal deaths are preventable, there are intersecting risk factors that have continued to put Black/African American women and their infants at risk for adverse pregnancy outcomes [5]. Experiences of racism, trauma, chronic stress, limited economic opportunities, and healthcare discrimination contribute to women’s reduction in use of perinatal health care [6]. These same factors impact pregnancy experience and health status and have been associated with increased risk for preterm and low birthweight babies [7, 8].

Feelings of hope and hopelessness are also integrated with women’s social and psychological circumstances, quality of perinatal care, and experiences during and after pregnancy [9–11]. The consequences for Black/African American women can include inequitable economic opportunities, inadequate healthcare, and experiences and resulting sequelae related to trauma and chronic stress. Data indicate that hope can be a protective factor for feelings of defeat and entrapment which are associated with risk for depression, anxiety, trauma, and suicidality [12–14].

Perinatal care that involves healthcare workers (HCWs) who are attentive and responsive to the needs of healthcare recipients (HCRs) can inspire feelings of hopefulness among women experiencing complicated pregnancies and postpartum conditions. Concurrently, meaningful and quality healthcare for women can support feelings of hopefulness among HCWs [15, 16].

### The hope initiative

The Hope Initiative was established as a collaboration between the Henry Ford Health Global Health Initiative (HFH GHI) in Detroit, USA and the Ihangane Project (TIP-GH) in Ruli, Rwanda. The partners were awarded a Robert Wood Johnson Foundation (RWJF) grant to co-design a project to explore the association of hopefulness and reduction of HCW burn out and improved health experiences and outcomes among pregnant and post-partum women.

At HFH GHI, the mixed-methods study explored the sources and impact of hope and hopelessness among HCWs and HCRs in two Women’s Health clinics in Metropolitan Detroit. Rates of Black/African American maternal and infant mortality in this area are among the highest in the state and well above the national average [17, 18].

The study aims were to: (1) increase knowledge and understanding of obstetric HCWs perceptions of hopelessness and hope and their experiences of patient care and impact on HCW burnout; (2) increase knowledge and understanding of Black/African American HCRs perceptions of hopelessness and hope and their experiences related to pre-natal, delivery, and post-partum care; (3) Identify HCW and HCR perspectives of strategies to reduce HCW burnout and improve the healthcare experiences of pregnant and post-partum Black/African American HCRs.

In March 2020 prior to project implementation at HFH, a two-day workshop was held in Detroit and included TIP-GH team members from Rwanda, GHI project staff, staff from collaborating HFH departments such as Women’s Health (WH), Behavioral Health Services (BHS), Community Health, Equity, Wellness and Diversity (CHEWD) and other stakeholders involved in maternal and infant health, healthcare worker well-being, and community outreach. Following the workshop, GHI and TIP-GH collaborated on project implementation in their respective settings, which included regular virtual meetings, mutual sharing of research instruments, and provision of technical assistance. In Detroit, GHI also worked closely with relevant HFH departments (BHS, WH, CHEWD).

A second joint GHI and TIP-GH workshop was held in Detroit at the conclusion of the project to present key findings and obtain feedback and recommendations from multiple stakeholders representing HFH, Wayne State University, local government, maternal and child health programs and community organizations.

### Conceptual framework

The study is based on the model of hope developed by Kaye Herth and operationalized in the Herth Hope Index survey (HHI) [19]. The HHI is based on a multidimensional definition of ‘hope’ which includes philosophical, religious, sociological and psychological factors. The HHI includes three constructs: temporality and future, positive readiness and expectancy, and interconnectedness. These constructs measure confidence and motivation toward the future, an acceptance of its uncertainty, and a capacity for relational interconnectedness that fosters a sense of meaning and well-being in one’s life [20]. Depression, anxiety, and hopelessness are negatively correlated with these factors.

In addition, experiences of HCWs and pregnant/postpartum HCRs are also influenced and impacted by wider socioeconomic and structural elements, both within communities and health systems [21, 22]. Therefore, these broader contextual factors were incorporated into the study design including items on the qualitative instruments about racism, discrimination in healthcare and services, and other social determinants affecting HCWs and HCRs experiences.

## Research methods

The mixed methods exploratory research included: (1) quantitative demographic data and the HHI instrument; and (2) qualitative focus group discussions and individual interviews. In addition, a comprehensive literature review was completed prior to the development of the qualitative research instruments. Two workshops were conducted before and after project implementation.

### Workshop 2020

The March 2020 workshop at HFH brought together approximately 30 stakeholders from a wide variety of institutions including hospitals (HFH Departments of Women’s Health and Behavioral Health, Wayne State Medicine, Detroit Medical Center), funders (Robert Wood Johnson Foundation), community based and global health organizations (HFH Global Health Initiative, TIP-GH), and educational institutions (e.g. John Hopkins University). The group included experts in medicine, nursing, midwifery, psychiatry, education, social sciences, as well as administrators, program managers, and foundation relation representatives. Participants worked in small groups (6 to 8 individuals) to discuss key questions to guide the development of the Hope Initiative. Together, participants sought to answer the following questions which would guide the development of the Hope Initiative intervention in Detroit: (1) What does a thriving Detroit look like? (2) What factors inspire hopefulness among health care workers (HCW)? (3) What factors diminish hope for HCW? (4) What factors inspire hopefulness for health care recipients (HCR)? (5) What issues diminish hope for HCR?

Outcomes included identification of factors that inspire hopefulness or diminish hope for both HCWs and Black/African American pregnant and postpartum HCRs.

### Literature review

A literature review was undertaken by the GHI team to understand how hopefulness mediates healthcare experiences of pregnant and new mothers, and their health care providers. Key insights from the literature informed the content of interview guides for focus groups and individual interviews.

The review identified challenges and sources of stress, as well as forms of support, which affected HCWs feelings of hope while providing care. It also reflected sources of stress and support for HCRs while receiving perinatal care which influenced their feelings of hope during their pregnancy and post-partum [9, 23].

### Research setting

The research setting included two HFH Women’s Health clinics in the metro Detroit area, serving primarily Black/African American women. In 2020–2021, 1,144 Black/African American women delivered from the larger study clinic and 233 Black/African American women delivered from the second clinic. Most of the research was done virtually.

### Participant recruitment

Eligible HCRs were identified using a data query from the HFH electronic medical record and included women who were pregnant at the time of the study, or who had delivered between 2020 and 2023 and received prenatal care at either of the two clinics. Additional criteria included race (Black/African American) and age (between 18 and 50). Exclusion criteria included having delivered in the past three months or being due to deliver in the next three months. Women included in the resulting query were contacted by email using recruitment materials developed by the team with input from the CHWs included in the project.

The HCRs received an incentive in the form of a gift card (US$30) for participation and were invited to the dissemination workshop to receive the results of the study.

HCWs who worked in the study clinics at the time of the study were contacted via email and asked to attend scheduled focus groups or offered the opportunity for an individual interview. They were also made aware of the study at staff meetings and through communications from leadership. Participation was voluntary.

Participants who expressed interest in a focus group/interview were sent a link which enabled them to complete the consent form, a brief demographic survey, and the HHI instrument.

### Quantitative methodology

#### Instruments

##### Demographics

The HCW demographic form included gender, ethnicity, age, employment status, position, and education. The HCR demographic form included information on gender, sexual orientation, age, religion, marital status as well as a brief section on socio-economic data and history of pregnancies. The HCR demographic form was developed in coordination with HFH CHWs engaged with programs for pregnant and post-partum women.

##### Herth Hope Index (HHI)

The standard HHI instrument was used including the three subscales: (1) temporality and future; (2) positive readiness and expectancy; and (3) interconnectedness. Each subscale included four items with four response options [strongly disagree, disagree, agree, strongly disagree]. High scores indicate more hopefulness. (see Table 1)

**Table 1:** Herth hope index: items and subscales

Temporality and Future	I have a positive outlook toward life (item 1)
	I have short and/or long-term goals (item 2)
	I feel scared about my future* (item 6)
	I believe each day has potential (item 11)
Positive Readiness	I can see possibilities in the midst of difficulties (item 4)
	I can recall happy / joyful times (item 7)
	I have a sense of direction (item 10)
	I feel my life has value and worth (item 12)
Interconnectedness	I feel all alone * (item 3)
	I have a faith that gives me comfort (item 5)
	I have deep inner strength (item 8)
	I am able to give and receive caring/love (item 9)

*Items 6 and 3 were reverse scored in the analysis

#### Data collection, management, and analysis

Data from HCWs was collected in 2021 and 2023. Data from HCRs was collected in 2023. Consent, demographic and HHI data were collected and managed using REDCap electronic data capture tools hosted at Henry Ford Health [24, 25]. REDCap (Research Electronic Data Capture) is a secure, web-based software platform designed to support data capture for research studies, providing (1) an intuitive interface for validated data capture; (2) audit trails for tracking data manipulation and export procedures; (3) automated export procedures for seamless data downloads to common statistical packages; and (4) procedures for data integration and interoperability with external sources. Both demographic and HHI data were downloaded into Excel and migrated into SPSS V. 25 (IBM). Data were reviewed to identify missing data and outliers. Within the HHI data sets, variables were created to enable comparison across both the complete HHI and within each subscale.

Descriptive data analysis included frequencies and mean scores with standard deviations and range. Bivariate analysis included cross tabulation Pearson’s chi-square for categorical data and independent samples tests for continuous data to assess significance between data sets. Cronbach’s alpha was calculated for the HHI scale and subscales to determine internal consistency for both the healthcare provider and recipient populations.

### Qualitative methodology

#### Instruments

To explore the impact of hopefulness for HCWs and HCRs semi-structured interview guides were created based on the literature review, perspectives of the kick-off workshop participants, and the constructs in the HHI. The HFH team also incorporated learnings from the qualitative data collected by TIP-GH. Draft interview guides were vetted by the HFH CHWs for language and cultural appropriateness.

##### Health care workers

The HCW interview guide included questions about 1) Understanding and defining hope and hopelessness 2) Hope in the work setting (e.g. feelings at work, work relationships/interactions, workplace infrastructure, and burnout), and 3) Patient-provider relationships (e.g., experiences of patient - provider positive and negative interactions)..

##### Health care recipients

The HCR interview guide included questions about (1) Understanding and defining hope and hopelessness and (2) Hope in health care settings (e.g., positive and negative experiences during pre/postnatal care, feelings of respect and disrespect in health settings, and social support systems).

#### Data collection, management and analysis

Focus groups and individual interviews were audio-taped and transcribed. Experienced qualitative researchers conducted and supervised the data collection, management, and analysis. Analysis and interpretation of the data included the full research team and subsequently participants at the post-implementation workshop. Transcribed data were uploaded into Dedoose, a web-based qualitative data management program [26]. This program provides simultaneous access to the data by multiple team members.

A content analysis approach was used and included an interpretive and naturalistic approach focused on the narrative data. A coding dictionary was developed based on the interview guide, study objectives, and emergent issues. An iterative process was used resulting in some codes being modified or added during the coding. Regular team meetings were held throughout data management, analysis, and interpretation. After coding, themes were identified within the coded texts and analyzed data were separated into tables by codes and themes. Final data tables included codes, themes, representative quotes from the texts and the recommendations generated by the study participants.

### Workshop 2024

The workshop took place at HFH. The first day was a smaller organizational meeting to review GHI and TIP-GH presentations and obtain collaborative input on finalizing content and delivery prior to the Day 2 Workshop. The Day 2 Workshop was designed to provide a forum for GHI and TIP-GH to share their research findings to a broad representation of stakeholders from HFH Women’s Health, Wayne State Health, the Detroit Health Department, and local community-based organizations supporting Black/African American women’s healthcare during and after pregnancy. Participants were divided into four small groups (8 to 10 individuals). Each group reviewed the findings from the GHI project and prioritized the most critical issues affecting hope and hopelessness among HCRs and HCWs. Each group generated recommendations for interventions to support hope for HCRs and HCWs and improve access to quality care.

## Results

### Workshop 2020

Participants generated factors affecting hope in both HCRs and HCWs and potential solutions to reduce burnout and improve pre- and post-partum care for Black/African American women. Participants provided guidelines for interventions and desired outcomes with relevant questions and considerations.

### Quantitative data

#### Demographics healthcare workers

In 2021, 39 healthcare workers completed the demographic form and HHI. In 2023, 27 providers completed the forms. Ten HCWs participated in both years. In 2021 and 2023, a subset of 34 (87.2%) and 25 (92.6%) HCWs participated in focus groups or interviews, respectively. In both years, a vast majority of participants were female and full-time employees of HFH. In 2021, the largest percentage of respondents were White [16 (41.0%)]; in 2023, the largest percentage were Black/African American [(14 (51.9%)]. In 2021 compared to 2023, more medical residents participated in the study [13 (33.3%) vs. 2 (7.4%) and there were more respondents with graduate degrees [24 (61.5%) vs. 8 (29.6%)]. However, there were no significant differences in demographic characteristics between the two data collection points. (see Table 2)

**Table 2:** Healthcare provider demographic data 2021 and 2023

		2021 participants *N* = 39	2023 participants *N* = 27	Total participants *N* = 66	
Category	Response	Outcomes			*p* value
Gender	Woman	35 (89.7%)	26 (96.3%)	61 (92.4%)	0.323
Ethnicity	White	16 (41.0%)	10 (37.0%)	26 (39.4%)	0.483
	Asian	5 (12.8%)	1 (3.7%)	6 (9.1%)	
	Black/African American	14 (35.9%)	14 (51.9%)	28 (42.4%)	
	Latino(a)	3 (7.7%)	2 (7.4%)	5 (7.6%)	
	Middle Eastern	1 (2.6%)	0	1 (1.5%)	
Age (Mean, SD)		38.2 (SD 12.6)	41.7 (SD 12.6)		0.826
Employment Status	Full Time	36 (92.3%)	25 (92.6%)	61 (92.4%)	0.966
Position	Physician	3 (7.7%0	5 (18.5%)	8 (12.1%)	0.081
	Resident	13 (33.3%)	2 (7.4%)	15 (22.7%)	
	Nursing	10 (25.6%)	11 (40.7%)	21 (31.8%)	
	Midwife	2 (5.1%)	0	2 (3.0%)	
	Medical Assistant	10 (25.6%)	9 (33.3%)	19 (28.8%)	
	Other	1 (2.6%)	0	1 (1.5%)	
Education (highest)	High school diploma or equivalent(GED)	2 (5.1%)	1 (3.7%)	3 (4.5%)	0.065
	College, no degree	4 (10.3%)	5 (18.5%)	9 (13.6%)	
	Undergraduate	9 (23.1%)	13 (48.1%)	22 (33.3%)	
	Graduate	24 (61.5%)	8 (29.6%)	32 (48.5%	

#### Demographics and healthcare recipient pregnancy data

In 2023, 61 healthcare recipients completed the demographic form and HHI. A subset of 27 (44.3%) of those individuals participated in focus group discussions. All participants were female, and a majority identified as heterosexual (84.2%). Over 98.2% identified as Black/African American. Most respondents reported their highest level of education as high school diploma/GED or some college (68.9%) and were employed full- or part-time (65.3%). Respondents reported either employee-based insurance or Medicaid. Demographic data of HCR who participated in focus group discussions (FGD) were compared to those who did not participate to identify any variation in FGD participation. Level of education was the only significant difference in demographic data between those who participated in focus group discussions and those who did not participate. (see Table 3)

**Table 3:** Healthcare recipient demographic data for FGD participants, non-participants, and total participants

		FGD participants *N* = 25	Non-participants *N* = 32	Total participants *N* = 57	
Category	Response	Outcomes			*p* value
Gender	Woman	25 (100%)	32 (100%)	57 (100%)	N/A
Sexual Orientation	Heterosexual	21 (84.0%)	27 (84.4%)	48 (84.2%)	0.993
	Bisexual	2 (9.4%)	3 (8.0%)	5 (8.8%)	
	Asexual	1 (4.0%)	1 (3.1%)	2 (3.5%)	
	Prefer to Self-Describe	1 (4.0%)	1 (3.1%)	2. (3.5%)	
Ethnicity	Black/African American	25 (96.2%)	31 (100%)	56 (98.2%)	0.271
	Other	1 (3.8%)	0	1 (1.8%)	
Age (Mean, SD)*		32.1 (SD 5.0) 25 to 42	31.8 (SD 6.4)	32.0 (SD 5.7)	0.916
Education	Some high school, no diploma	2 (8.0%)	0	2 (3.4%)	0.049
	High school diploma or equivalent(GED)	6 (24.0%)	14 (42.4%)	20 (34.5%)	
	Some college	6 (24.0%)	12 (36.4%)	18 (31.0%)	
	Trade/vocational	0	3 (9.1%)	3 (5.2%)	
	Associate degree	3 (12.0%)	1 (3.0%)	4 (6.9%)	
	Bachelor’s degree	6 (24.0%)	2 (6.1%)	8 (13.8%)	
	Post-grad/ professional degree	2 (8.0%)	1 (3.0%)	3 (5.2%)	
Currently in school	Yes	7 (29.2%)	4 (12.1%)	11 (19.3%)	0.107
Employment Status	Full Time	16 (61.5%)	14 (43.8%)	30 (51.7%)	0.163
	Part Time	5 (19.2%)	3 (9.4%)	8 (13.8%)	
	Unemployed, looking for work	1 (3.8%1)	10 (31.3%)	11 (19.0%)	
	Unemployed, not looking for work	1 (3.8%)	1 (3.1%)	2 (3.4%)	
	SAHM	0	2 (6.3%)	2 (3.4%)	
	Self-employed	1 (3.8%)	1 (3.1%)	2 (3.4%)	
	Student	1 (3.8%)	0	1 (1.7%)	
	Other	1 (3.8%)	1 (3.1%)	2 (3.4%)	
Marital Status	Single, Never Married	11 (45.8%)	19 (57.6%)	30 (52.6%)	0.283
	Married/Domestic Partner	12 (50.0%)	12 (36.4%)	24 (42.1%)	
	Separated	1 (4.2%)	0	1 (1.8%)	
	Divorce	0	2 (6.1%)	2 (3.5%)	
Health Insurance	Employee-based	14 (51.9%)	11 (33.3%)	25 (41.7%)	0.263
	Marketplace^a^	0	1 (3.0%)	1 (1.7%)	
	Medicaid	13 (48.1%)	21 (63.6%)	34 (56.7%)	

^a^Marketplace refers to purchasing private health insurance

*Only 23/61 (37.7%) participants provided age

Overall, 31.1% of participants were currently pregnant. Mean number of past pregnancies was 3.9 (SD 2.2) and mean age of first pregnancy was 23.1 years (SD 4.7). Mean number of children was 2.1 (SD 1.9). Mean number of deliveries either under 37 weeks or over 42 weeks was less than one. There were no significant differences between women who participated in the focus group discussion and those who did not participate (see Table 4).

**Table 4:** Healthcare recipient pregnancy history data for FGD participants, Non- participants, and total participants

		FGD participants	Non-participants	Total participants	
Category	Response	Outcomes			*p* value
Currently pregnant	Yes	9 (33.3%)	10 (29.4%)	19 (31.1%)	0.743
Age first pregnancy		23.0 (SD 4.7)	23.1 (SD 4.8)	23.1 (SD 4.7)	0.981
Lifetime number pregnancies		4.3 (SD 2.0)	3.5 (SD 2.3)	3.9 (SD 2.2)	0.195
Delivery under 37 weeks		0.44 (SD 0.80)	0.76 (1.2)	0.62 (SD 1.0)	0.233
Delivery over 42 weeks		0.19 (SD 0.56)	0.09 (SD 0.29)	0.13 (SD 0.43)	0.383
Total number of children		2.4 (SD 2.1)	1.9 (SD 1.8)	2.1 (SD 1.9)	0.235

#### Herth hope index scores for healthcare workers and healthcare recipients

Overall, HCWs scored high on both the total HHI scale and the three subscales. The 48-point HHI mean score was 41.7 (SD 4.6). The 16-point subscale means were 13.6 (SD 1.2) for temporality and future, 14.2 (SD 1.5) for positive readiness and expectancy, and 13.9 (SD 1.9) for interconnectedness. The Cronbach’s alphas were over 0.70 for the total HHI and positive readiness and expectancy and interconnectedness. There were no significant differences in scores between the 2021 and 2023 participants (see Table 5).

**Table 5:** Herth hope index scale & subscales with mean scores (SD), *p* values, and Cronbach alpha [HEALTHCARE WORKERS]

	2021 participants	2023 participants	Total participants	*p* value	Cronbach’s alpha
Total HHI	41.5 (SD 4.7)	42.0 (SD 4.6)	41.7 (SD 4.6)	0.716	0.89
Temporality and Future	13.7 (SD 1.5)	13.6 (SD 1.8)	13.6 (SD 1.6)	0.857	0.61
Positive Readiness and Expectancy	14.2 (SD 1.6)	14.3 (SD 1.5)	14.2 (SD 1.5)	0.836	0.77
Interconnectedness	13.7 (SD 1.9)	14.1 (SD 1.8)	13.9 (SD 1.9)	0.371	0.71

Ranges: HHI 12 to 48; Subscales 4 to 16

HCRs also scored high on both the total HHI scale and the three subscales. The 48-point HHI mean score was 40.8 (SD 7.0). The 16-point subscale means were 13.5 (SD 2.4) for temporality and future, 13.5 (SD 2.6) for positive readiness and expectancy, and 13.7 (SD 2.4) for interconnectedness. The Cronbach’s alphas were over 0.70 for the total HHI and the three subscales. There were no significant differences between those who participated in the focus group discussions and those who did not participate (see Table 6).

**Table 6:** Herth hope index scale & subscales with mean scores (SD), *p* values, and Cronbach alpha [HEALTHCARE RECIPIENTS]

	FGD participants	Non FGD participants	Total participants	*p* value	Cronbach’s alpha
Total HHI	40.3 (SD 9.0)	41.1 (SD 5.0)	40.8 (SD 7.0)	0.627	0.94
Temporality and Future	13.6 (SD 3.0)	13.5 (SD 1.9)	13.5 (SD 2.4)	0.847	0.79
Positive Readiness and Expectancy	13.2 (SD 3.3)	13.8 (SD 1.9)	13.5 (SD 2.6)	0.399	0.87
Interconnectedness	13.4 (SD 3.0)	13.9 (SD 1.8)	13.7 (SD 2.4)	0.482	0.82

Ranges: HHI 12 to 48; Subscales 4 to 16

In addition to use of the Cronbach’s alpha scores for internal consistency/reliability, we also matched responses from the qualitative interviews for each scale item. Through this process, the HHI scale was determined to identify key elements of hopefulness and hopelessness as expressed by both HCWs and Black/African American HCRs. In future studies, the qualitative data will be analyzed to determine if there are constructs missing that could provide a more accurate measure for these study populations [27]. 

### Qualitative data

The qualitative data includes themes and representative quotes from both HCWs and HCRs. Key outcomes from the HCWs include: (1) defining hopefulness and hopelessness: (2) sources of care and support; (3) patient needs and experiences; and (4) health system challenges. Key outcomes from the HCRs include: (1) defining hopefulness and hopelessness; (2) sources of care and support; and (3) pregnancy and post-partum healthcare experiences and outcomes. The outcomes reflect the responses to questions specifically targeting hopefulness and hopelessness and questions focused on the contextual factors and related positive and negative experiences that impact hope.

#### Health care workers

#### Defining hopefulness and hopelessness

Respondents were asked to define both hopefulness and hopelessness. Respondents described hopefulness as looking forward and the possibility of positive change through themselves and/or their children. They also mentioned acknowledging past progress, having a support system, and faith.

*…hope is having a feeling that your future goals can be met*,* feeling optimistic about joyfulness in your life and that if things are challenging*,* that things will change…* (Midwife 2021).

*…my kids make me feel hopeful just because of the potential that they have…* (Medical Assistant, 2023).

…*looking towards the future*,* learning from the past*,* and my faith…keeps me together”* (Medical Assistant, 2021).

Respondents described hopelessness as feelings of lack of control, relations, and inability to make changes.

*………You feel like no matter what you do*,* you aren’t going to have an impact…* (Midwife 2021).

…*Feeling lost in the world…you don’t feel love. You don’t feel like there’s a future…* (Provider, 2021).

#### Sources of care and support

HCW described a broad range of factors that enable them to feel hopeful or to mitigate feelings of hopelessness. This included self-care, relationships with family, peers and colleagues, and clinical practice. They also described aspects of their life and work that increased feelings of hopelessness.

##### Self-care and spirituality

In terms of self-care, HCWs described the importance of having a positive outlook. Spirituality and faith were considered important sources of support and renewal in difficult times.

*I may be in a situation where I’m like*,* “Okay*,* this is just hopeless.” Then what I have to do is regroup*,* sit myself down and get out of that space and say*,* “Okay*,* you know what? Nothing is hopeless. Let me think about*,* how do I reposition and start all over? ” Because as long as I keep breathing*,* I can always start all over…* (Nurse 2021)​

*I’ve always gone to church…So when things are bad*,* I can go to my favorite Bible verse*,* or I can call my dad…he’ll pray (and) just to bring in a new spirit within me* (Medical Assistant 2021).

HCWs recommended approaches to support self-care adequate time for self-care activities, and a space at work to relax and reduce stress.

##### Family support

Family was an important source of support and hopefulness for HCWs.

*My family is a big support for me*,* and I think that gives me a lot of hope for having the life I hope to achieve that makes me happy and fulfilled* (Midwife 2021).

*Sometimes work gets a little too stressful*,* so you got to look for the hopefulness at home*… (Nurse 2023).

HCWs described their need to have more flexible work hours and a better life-work balance to take care of personal and family needs.

*I think that if you’re working consistently more than 50 to 60 hours a week*,* that should be frowned upon. That should be looked at and be like*,* “This is not healthy for anyone.” I mean*,* granted I understand that it’s a rite of passage as a physician*,* but I thought it was a passage and not just the*,* “This is what my life is.” So I think that maybe limiting the amount of hours that the physicians and the midwives have to work could create a healthier work-life balance.* (Physician 2021)

#### Work-based support systems

HCWs appreciated having an outlet to discuss challenges with peers and colleagues who also provided motivation and support.

*I do have an extensive network of my fellowship colleagues…I’ll reach out to them so they can just listen to me vent…* (Physician 2023)

*I appreciate my coworkers for checking up on me*,* and for (their) understanding because we have different gifts…* (Medical Assistant 2021).

HCWs recommended the importance of having an interdisciplinary team which meets regularly and incorporates team building exercises. Within their clinical practice, HCWs noted that responsive leadership provided an important source of affirmation. HCWs also requested opportunities to share their experiences and issues in delivering health care with a third party. Patients’ acknowledgement of the HCW also supports feelings of hope.

*…Knowing that we had that leadership there to support us… and not talk at us*,* but with us*,* has been one thing that helps* (Medical Assistant 2021)

*I’ve had some patients that after a visit they’re like*,* “Thank you so much for listening to me because I’ve been to multiple doctors*,* and no one has taken the time to sit down and listen to me or taken the time to give me options.”* (Physician 2023).

HCWs recommended having self-support mechanisms to balance positive and negative experiences in their work. HCWs requested opportunities to continue their training and implementation of innovative approaches to pregnancy and delivery to improve patient outcomes and satisfaction in their work.

#### Patient needs and experiences

HCWs noted that HCRs who lack a social support system or are in abusive relationships have multiple needs. However, resources are often inadequate or inaccessible. HCWs also discussed experiences when patients do not receive the support needed, despite the best efforts of the clinician.

*…we’ve had a young girl living by herself pregnant. She won’t turn to her parents because they won’t support her…She lost her job because of her hyperemesis*,* her nausea and vomiting…She’s struggling…* (Nurse 2023).

*…when a patient gets comfortable with you*,* and they share their life with you… You can provide as much assistance as the clinic has…sometimes that may not be enough…So*,* for me*,* that would be like*,* “I can’t do anything.”* (Medical Assistant 2021).

HCWs recommended increases in staff to meet patient needs including a dedicated person, e.g., nurse navigator, to address personal and social needs. HCWs also discussed the need for diversity in staff to better service Black/African American HCRs.

#### Health system challenges

Policy changes related to staffing and scheduling are decreasing continuity of care and thereby limiting opportunities for HCWs to establish trusting relationships with patients.

*Our clinics weren’t really (providing) continuity*,* and you were seeing different patients all the time. Patients were made to wait an hour plus to see you…in general experiences were negative because their expectations were not met* (Resident 2021).

#### Health care recipients

#### Defining hopefulness and hopelessness

HCRs defined hopefulness as trust in the future and striving for positive outcomes. Hopelessness was defined as feelings of discouragement, depression, anxiety, and loneliness.

*When I think of the word hopefulness*,* it kind of gives me inspiration. Something like to look forward to….almost like faith attached to it….*

*Hopelessness is when someone is just feeling defeated and feeling like they don’t have a different avenue or a different route to do what needs to be done….*

#### Sources of care and support

##### Self-care

HCRs described the importance of taking care of one’s health and fitness. They also described education, family, and spirituality as key sources of self-care.

*In prenatal care*,* I feel like if you’re hopeful…you’re going to do everything that you can so that you’re capable to make sure that your baby is as healthy as possible*,* that you’re as healthy as possible…watching the food you eat…doing a little bit of exercise*. (HCR 2023) *My kids*,* especially by them being older*,* really look up to me and see how hard I work for them…I have to go an extra mile just so they can see*,* especially by them being young*,* Black men*,* and what’s going on in the world.* (HCR 2023)

##### Community support systems

HCRs also discussed obtaining support through friends and community groups. HCRs identified sharing experiences with other women who have pregnancy and post-partum experiences.

*I do believe it takes a village …Yeah*,* its family*,* friends*,* neighbors*,* it’s that being able to be social.* (HCR 2023)

*Just anyone who can kind of encourage me*,* motivate me*,* someone who’s been in my position*,* who understands what I’m going through and can encourage me as well that it will get better.* (HCR 2023)

HCRs recommended that Black/African American pregnant women and new mothers have opportunities to share their experiences and information with each other to increase their confidence. HCRs requested that resources for new mothers, such as daycare, be continued to help care for their children and support their work life.

#### Pregnancy and post-partum healthcare experiences and outcomes

##### Racism and discrimination

Black/African American HCRs discussed bias, racism, and disparities within their healthcare experiences. HCRs mentioned feeling dismissed and not being listened to by healthcare providers. Alternatively, some respondents did not feel they had experienced racism.

*I think race plays a significant role… As if*,* we have this invincibility for pain as Black people and honestly we do not….* (HCR 2023)

*Some of them might be rude. Sometimes I be a little rude back*,* but that’s a different story. But I don’t really feel like it’s a really race thing because that’s like if every time something going on you be like*,* “Oh*,* because they white….”* (HCR 2023)

##### Continuity of care and HCW-HCR relationships

Continuity of care was also an issue which contributed to a lack of trust in their provider and/or the health system.

*It is difficult when you’ve seen the same doctor the first trimester of your pregnancy and then someone comes in*,* a total stranger…You don’t have a choice…and it’s not always to our liking….*(HCR 2023)

##### Self-advocacy

HCRs described the importance of having a voice in decision-making and positive experiences within the health system to decrease stress and anxiety.

…*the doctor made sure everything was good. Her nurse made sure that everything was good… But that doctor the prior week… didn’t understand or didn’t care…that goes back to the stress-free and happy baby.* (HCR 2023)

*Because I have that feeling that my baby is about to come. They listened to me*,* checked my cervix and it was ten centimeters*,* and I was able to push my baby out…I was able to have a healthy baby.* (HCR 2023)

HCRs recommended that HCWs listen to women’s experiences and be actively engaged in their care. They also emphasized the importance of continuity of care, communication, and trust. Some HCRs also recommended that clinic staff reflect the ethnic and racial characteristics of the community they serve.

### Workshop 2024

Workshop participants selected one of the presented themes and/or recommendations and discussed potential interventions based on their selection (see Table 7). They also identified the stakeholders to be involved in implementing the proposed intervention, the resources needed and potential barriers.

**Table 7:** Workshop participant selections of themes and recommendations

Group	Category	Theme	Recommendation
1	HCW-Self-Care	Positive approach to work and place in Material Child Health	Opportunities for HCWs to discuss perspectives with a third party
2	HCW-Health System	Insufficient resources for patients	N/A
3	HCW-Patient needs & Experiences	Systemic lack of resources/access to resources for patients	Dedicated person to address needs of patients, (e.g., nurse navigator)
4	HCR-Peers & Community	Support from HCW, peers, and partners	Opportunities for Black/African American pregnant women and new mothers to share their experiences with peers, have access to doulas and midwives. Need for support programs for fathers

Group 1 discussed opportunities for sharing perspectives and grievances with a third party who would listen and respond to HCW concerns. The workshop participants suggested having a dedicated person as a liaison between the HCWs and the administration to alleviate stress and risk for burnout and staff turnover. The group identified an existing position at HFH which could be appropriate for this role.

Group 2 and 3 both discussed the lack of resources for patients with multiple needs. One option suggested by participants was to establish coordination of available resources. They suggested creating an information hub, such as a website, where information would be centralized, updated and readily available to the public. Another solution focused on creating a position such as a nurse navigator who would be dedicated to developing a resource hub and assisting patients identify and access resources. During discussions, existing resource hubs were identified which could be used or adapted to meet the needs of pregnant and post-partum patients.

Group 4 participants discussed the importance of available support systems during pregnancy and post-partum including peer support and accessible midwives and doulas. The group further highlighted the need for similar support and outreach services for fathers. Existing and new programs were discussed which could be expanded or adapted to meet the expressed needs including an on-going fatherhood support group.

During the second workshop, the participants prioritized interventions (see Table 7). From these selected interventions, the research team and stakeholders prioritized the need for inclusion of fathers in outreach and educational efforts and the importance of having an independent dedicated individual as a liaison to support communication between HCWs and the health system. These interventions were further discussed and developed with the respective HFH departments and are awaiting implementation.

In terms of implementation of these recommendations, workshop participants discussed the need for HFH leadership support and interdepartmental and interagency collaborations, financial commitment, and sufficient human resources.

## Discussion

Previous research has shown that hope can encourage health-promoting behaviors and lead to better outcomes [28]. This study explored the influence of hopefulness on the use of health care by pregnant and post-partum Black/African American women and HCWs delivery of quality obstetric care in Women’s Health clinics in Detroit, Michigan.

Findings indicate that both HCWs and HCRs scored high on both the total HHI scale and the three subscales. In the qualitative interviews, HCRs and HCWs discussed both situations that support hopefulness and those that increase feelings of hopelessness and can lead to stress, anxiety, and burnout.

HCWs spoke about the importance of hopefulness in terms of the health and well-being of both women and infants. Recent studies demonstrate the association of hope in relation to sustained and adequate perinatal care. These studies also identify social factors which can contribute to hopelessness and result in poor health outcomes [16, 29]. Similar to other research, HCRs associated hopefulness with support from family and friends, their relationship with their children, self-improvement, and spirituality [30]. Furthermore, HCRs described the importance of work-life balance, their physical and mental health, and the well-being of their babies as key factors associated with hopefulness.

Among HCWs, one of the most reported challenges was lack of access to resources and referrals needed by their patients. This has been a prominent issue in other healthcare settings particularly those serving communities with limited resources. To address this need, literature supports interventions which focus on the development of resources at the local level [31]. HCWs indicated that not being able to address patients’ needs created feelings of hopelessness and risk for burnout, which has been described in a number of studies [32]. HCWs discussed the need for strategies to support their ‘self-care’, along with being able to meet their personal and professional goals and responsibilities. They also described the importance of being able to express their views with leadership. However, the HCWs also stated that feedback was seldom translated into policies or programs. Literature on moral distress among HCWs indicate the importance role of leadership and development of policies which can support HCWs in situations where they know the ‘right’ course of action but are limited by institutional constraints. Literature suggests making ethics education and resources available to HCWs including ethics debriefings and development of programs to develop ethics capacity. Such approaches can potentially alleviate some internal distress experienced by HCWs and decrease burnout and feelings of hopelessness [33, 34].

HCWs described a strong sense of teamwork within the clinics, with care team members serving as a reliable source of support and a mediating factor for burnout and feelings of hopelessness. HCWs also described the importance of positive relationships with HCRs. However, establishing these positive relationships can be hindered because of health system policies that do not support continuity of care and limit time for patient visits. HCWs also acknowledged the systemic changes that affect workload, especially increases in responsibilities, and the impact of these system requirements on their own well-being and patient care and outcomes. These considerations have been increasingly recognized by researchers and professional associations as critical issues in HCWs’ effective functioning and resilience against burnout [35–37].

HCRs identified positive healthcare experiences including knowing who was on their care team and consistency in care, especially during delivery. They also discussed the role of communication with HCWs such as receiving detailed information about procedures and outcomes and having their preferences acknowledged. A recent study reported significant relationships between positive health outcomes for women and their babies and person-centered maternal care defined as care “.respectful of, and responsive to, women’s preferences, needs, and values” [38].

HCRs described the importance of support from other Black/African American women who have pregnancy and post-partum experience as well as support from health professionals. The Birthing Beautiful Communities (BBC) program in Cleveland and Akron, Ohio include women’s health professionals (e.g., doulas) and are peer-led. BBC has been found to reduce stressors and support resiliency and self-advocacy for Black/African American women. Furthermore, the program has identified specific social determinants of health, e.g. transportation, housing, and provides access to resources to specifically address these issues [39, 40].

HCRs discussed negative experiences related to racial biases on the part of HCWs, specifically in terms of being provided with inadequate medications for pain relief and microaggressions in terms of communication. Some HCRs also expressed preference for care provided by Black/African American HCWs.

Data from this study and research referenced above indicates the need for health system and community resources to address both health and social needs of HCRs and processes for referrals to these resources. HFH has implemented system changes to address this issue such as the Women-Inspired Neighborhood Network [41, 42] and, more recently, the introduction of a Nurse Navigator position. These types of programs increase HCRs access to care and reduce poor health and social outcomes [31].

### Study limitations

The implementation of the study was delayed by the Covid-19 pandemic, which started one month after the initial workshop. Also, some of the study activities had to take place virtually because of the Covid-19 precautions.

Recruitment for this study took place via email, which may have excluded a portion of the target population – Black/African American women at risk of adverse pregnancy outcomes because of their socioeconomic status – who do not have regular access to digital technology. However, the demographic data for our study reflects a variety of respondents in terms of education and employment, indicating this limitation may have had a minor impact.

Following the workshop, the study team selected to explore the intervention focused on dedicating a third party to serve as a liaison between HCWs and Women’s Health leadership. While there was enthusiasm from leadership, to date the team has been unable to find a candidate to fill this position. Based on further discussions related to the support for women, the team developed a Fatherhood Initiative. The proposal was welcomed by leadership of HFH Community Health, however, due to funding constraints it has not yet been implemented.

## Conclusion

The current study contributes to literature regarding the role of hope as a contributing factor in the experiences of healthcare workers and of Black/African American women during pregnancy and post-partum. These factors include importance of social, family, spiritual, peer, and institutional support, continuity of care and development of positive relationships between healthcare workers and healthcare recipients, and recognition and amelioration of interpersonal and structural racism and discrimination within health systems. Through both qualitative research and workshops, recommendations at the community, clinical, and systems level were identified to address hopelessness and support positive experiences and outcomes for both HCWs and HCRs. Next steps include identifying resources to pilot and evaluate recommendations.

## Supplementary Information

Supplementary Material 1.

Supplementary Material 2.

Supplementary Material 3.

## Data Availability

The datasets used and/or analyzed during the current study are available from the corresponding author on reasonable request.

## Abbreviations

HCW(s): Healthcare worker(s)
HCR(s): Healthcare recipient(s)
HFH: Henry Ford Health
GHI: Global Health Initiative
TIP – GH: The Ihangane Project
RWJF: Robert Wood Johnson Foundation
WH: Women’s Health
BHS: Behavioral Health Services
CHEWD: Community Health, Equity, Wellness and Diversity
HHI: Herth Hope Index survey
CHW(s): Community Health Worker(s)
REDCap: Research Electronic Data Capture
