# Supplementary material for: Inspiring hope, confronting hopelessness: healthcare experiences of Black/African American pregnant and post-partum women and healthcare workers in Detroit, Michigan, U.S.A
Source: BMC Pregnancy Childbirth. 2026 Jan 27;26:179. doi: 10.1186/s12884-026-08666-5 (PMC12918345; doi:10.1186/s12884-026-08666-5)
Supplement: Supplementary file 2 — Supplementary Material 2. [file 12884_2026_8666_MOESM2_ESM.pdf]

HEALTH CARE PROVIDER DEMOGRAPHIC FORM

1. Age__________
2. Gender__________________________
3. Ethnicity
   (1) White____   (2) Black / African American____   (3) Latina____   (4) Asian______
   (5) Middle Eastern____ (6) Native American______   (7) Other___________________
4. Employment status
   (1) Full time_____   (2) Part time_____
5. Position (at HFHS)________________________________
6. Time worked at current location (years)___________________
7. Time worked at HFHS (years)__________________
8. Time worked in prenatal/postnatal care___________________
9. Highest Education/Degree________________

APPROVED

23-APR-2021

INSTITUTIONAL REVIEW BOARD

VERSION: 12 APRIL 2021
