# Supplementary material for: Inspiring hope, confronting hopelessness: healthcare experiences of Black/African American pregnant and post-partum women and healthcare workers in Detroit, Michigan, U.S.A
Source: BMC Pregnancy Childbirth. 2026 Jan 27;26:179. doi: 10.1186/s12884-026-08666-5 (PMC12918345; doi:10.1186/s12884-026-08666-5)
Supplement: Supplementary file 3 — Supplementary Material 3. [file 12884_2026_8666_MOESM3_ESM.pdf]

APPROVED
April 17, 2023
INSTITUTIONAL REVIEW BOARD

HOPE | Demographic Questionnaire | Version 1
Date: 4.13.23

1. What is your age?
   a. Open Text

2. Which option best describes your employment status?
   a. Employed Full-Time
   b. Employed Part-Time
   c. Unemployed, looking for work
   d. Unemployed, not looking for work
   e. On disability
   f. Other

3. Are you currently in school?
   a. Yes
   b. No

4. What is the highest level of education you have completed?
   a. No formal education
   b. 8th grade
   c. Some high school, no diploma
   d. High school graduate (or equivalent, e.g. GED)
   e. Some college, no degree
   f. Trade/technical/vocational training
   g. Associate Degree
   h. Bachelor's degree
   i. Post-graduate or professional degree

5. What race/ethnicity do you identify with (*mark all that apply*)?
   a. African American
   b. Black
   c. Continental African
   d. Afro Caribbean
   e. Afro Latina / Hispanic
   f. Other _________

6. What is your sexual orientation?
   a. Asexual
   b. Bisexual
   c. Gay
   d. Heterosexual or straight
   e. Lesbian
   f. Pansexual
   g. Queer
   h. Prefer to self-describe: ________

7. What is your gender identity?
    a. Male
    b. Female
    c. Transgender
    d. Non-binary / gender-fluid
    e. Prefer to self-describe: _______

8. What is your current marital status?
    a. Single, never married
    b. Married or domestic partnership
    c. Separated
    d. Divorced
    e. Widowed

9. What is your religion?
    a. Christianity
    b. Judaism
    c. Islam
    d. Hinduism
    e. Buddhism
    f. None
    g. Other ________

10. What option best describes the type of insurance you have?
    a. Employer-based insurance
    b. Insurance through the "Health Insurance Marketplace"
    c. Private insurance / self-pay
    d. Medicaid
    e. Uninsured
    f. Other

11. What is the Zipcode where you currently reside?
    a. Open text

12. (*If currently pregnant)* How many weeks are you into your pregnancy?
    a. Open text

13. How many times have you been pregnant?
    a. Open text

14. Of those times, how many resulted in a live birth?
    a. Open text

15. Age when you had your first child
    a. Open text

16. How many of your children were born before 37 weeks?
    a. Open text

17. How many of your children were born after 42 weeks?
    a. Open text

18. How many children do you currently care for (adopted, stepchildren, grandchildren, etc.) in the following age groups?
    a. Under 2 years _____
    b. 2 to 5 years _____
    c. 6 to 12 years _____
    d. 13+ years _____
