# Supplementary material for: Inspiring hope, confronting hopelessness: healthcare experiences of Black/African American pregnant and post-partum women and healthcare workers in Detroit, Michigan, U.S.A
Source: BMC Pregnancy Childbirth. 2026 Jan 27;26:179. doi: 10.1186/s12884-026-08666-5 (PMC12918345; doi:10.1186/s12884-026-08666-5)
Supplement: Supplementary file 1 — Supplementary Material 1. [file 12884_2026_8666_MOESM1_ESM.pdf]

**From:** Herth, Kaye A <kaye.herth@mnsu.edu>
**Sent:** Wednesday, June 23, 2021 1:52 PM
**To:** MacLean, Lisa <LMACLEA1@hfhs.org>
**Subject:** RE: Permission to Use the Herth Hope Index

**CAUTION CYBER RISK: EXTERNAL E-Mail. Do not click links or open attachments unless you recognize the sender, expected to receive this content, and you believe it to be safe. Delete this message; or forward the email to spam@ hfhs org if suspicious.**

Dear Dr. MacLean,
I appreciate your interest in the Herth Hope Index (HHI). I have attached a copy of the HHI, scoring instructions, and two reference lists I have compiled of published articles on hope primarily from the nursing disciplines.

You have my permission to use the HHI in your research project as you described in your email message to me. I would appreciate receiving a summary of your study upon completion.

Best wishes in your highly important research work.
Sincerely,
Kaye Herth, Ph.D., R.N., F.A.A.N.
Dean Emerita
Minnesota State University, Mankato
